# Supplementary material for: Plasma Concentrations of Neurofilament Light Chain Protein and Brain-Derived Neurotrophic Factor as Consistent Biomarkers of Cognitive Impairment in Alcohol Use Disorder
Source: Int J Mol Sci. 2023 Jan 7;24(2):1183. doi: 10.3390/ijms24021183 (PMC9866623; doi:10.3390/ijms24021183)
Supplement: Supplementary file 1 [file ijms-24-01183-s001.zip › ijms-2007799-supplementary.pdf]

SUPPLEMENTARY DATA

Supplementary Table S1. Correlation analysis according to addiction-related variables and NfL plasma concentrations.

VARIABLES			SUD group N=60	Correlation Analyses	
				**NfL [Rho (*p* value)]**	**BDNF [Rho (*p* value)]**
**Age at first drug use** *[Mean (SD)]*	*Years*	*Alcohol*	14.04 (4.31)	**0.315 (0.031)**	-0.079 (0.651)
		Cocaine	19.22 (5.05)	**0.437 (0.008)**	-0.103 (0.640)
		Cannabis	14.60 (2.48)	0.170 (0.416)	-0.146 (0.603)
		Sedatives	18.50 (1.77)	0.491 (0.217)	0.103 (0.870)
		Opioids	20.86 (8.44)	0.306 (0.504)	0.600 (0.285)
**Age at onset of SUD** *[Mean (SD)]*	*Years*	*Alcohol*	17.85 (9.31)	**0.455 (0.001)**	-0.229 (0.189)
		Cocaine	24.75 (8.38)	0.304 (0.071)	-0.189 (0.387)
		Cannabis	16.92 (4.04)	0.144 (0.493)	-0.162 (0.565)
		Sedatives	24.25 (13.46)	0.596 (0.119)	-0.447 (0.450)
		Opioids	22.14 (7.99)	0.252 (0.585)	0.564 (0.322)
**Length of SUD diagnosis** *[Mean (SD)]*	*Years*	*Alcohol*	16.70 (12.06)	**0.375 (0.010)**	-0.229 (0.192)
		Cocaine	10.93 (8.65)	0.251 (0.140)	-0.104 (0.638)
		Cannabis	13 (10.01)	0.231 (0.266)	-0.005 (0.985)
		Sedatives	7.88 (5.94)	0.217 (0.606)	-0.053 (0.933)
		Opioids	14.57 (13.54)	0.198 (0.670)	-0.500 (0.391)
**Severity criteria** *[Mean (SD)]*	*Criteria [1-11]*	*Alcohol*	6.35 (2.91)	0.032 (0.831)	0.106 (0.543)
		Cocaine	6.11 (3.66)	-0.246 (0.148)	0.087 (0.694)
		Cannabis	5.73 (3)	-0.383 (0.059)	**0.605 (0.017)**
		Sedatives	7 (2.98)	-**0.815 (0.014)**	0.359 (0.553)
		Opioids	4 (4.36)	-0.299 (0.514)	-0.100 (0.873)
**Length of abstinence** *[Mean (SD)]*	*Days*	*Alcohol*	400.24 (995.31)	0.121 (0.422)	-0.163 (0.358)
		Cocaine	216.67 (672.13)	0.250 (0.141)	0.133 (0.546)
		Cannabis	526.68 (1488.22)	0.159 (0.447)	-0.094 (0.739)
		Sedatives	72.43 (167.31)	0.729 (0.063)	-0.632 (0.368)
		Opioids	1103.43 (1290.84)	0.500 (0.253)	-0.105 (0.866)

Supplementary Table S2. Analysis of plasma concentrations of NfL according to comorbid psychiatric disorders and psychotropic medication in the SUD group

VARIABLE		Total NfL (pg/ml) Mean [95%CI]	Statistics		
			F-value	df	*p*-value
**Psychiatric disorder**	No (N=14)	7.641 [6.083-9.199]	3.192	1,57	0.079
	Yes (N=46)	9.746 [8.896-10.595]			
**Major depressive disorder**	No (N=29)	9.132 [8.011-10.252]	0.016	1,57	0.900
	Yes (N=31)	9.369 [8.286-10.453]			
**Anxiety disorder**	No (N=39)	8.639 [7.710-9.568]	4.895	1,57	**0.031**
	Yes (N=21)	10.398 [9.123-1.672]			
**Psychotropic medication**	No (N=9)	8.845 [0.998-6.847]	0.015	1,57	0.901
	Yes (N=51)	9.327 [8.487-10.166]			
**Antidepressants**	No (N=33)	9.045 [8.009-10.082]	0.013	1,56	0.911
	Yes (N=26)	9.333 [8.165-10.500]			
**Anxiolytics**	No (N=25)	8.822 [7.638-10.007]	0.049	1,56	0.825
	Yes (N=34)	9.429 [8.414-10.445]			

Supplementary Table S3. Analysis of plasma concentrations of BDNF according to comorbid psychiatric disorders and psychotropic medication in the SUD group.

VARIABLE		Total BDNF (pg/ml)	Statistics		
		Mean [95%CI]	F-value	df	*p*-value
**Psychiatric disorder**	No (N=10)	2.949 [-1.527-7.424]	1.339	1,39	0.254
	Yes (N=32)	5.897 [3.423-8.327]			
**Major depressive disorder**	No (N=20)	1.542 [3.448-9.685]	0.673	1,39	0.417
	Yes (N=22)	3.948 [0.976-6.921]			
**Anxiety disorder**	No (N=29)	3.988 [1.410-6.565]	3.011	1,39	0.091
	Yes (N=13)	7.889 [3.975-11.802]			
**Psychotropic medication**	No (N=6)	5,113 [-0.671-10.898]	0.434	1,39	0.514
	Yes (N=36)	5.209 [2.848-7.570]			
**Antidepressants**	No (N=25)	4.905 [2.071-7.739]	0.090	1,39	0.766
	Yes (N=17)	5.622 [2.182-9.062]			
**Anxiolytics**	No (N=16)	4.287 [0.764-7.810]	0.464	1,39	0.500
	Yes (N=26)	5.754 [2.991-8.517]			

Supplementary Table S4. Analysis of NfL/BDNF rate according to comorbid psychiatric disorders and psychotropic medication in the SUD group.

VARIABLE		NfL/BDNF rate	Statistics		
		Mean [95%CI]	F-value	df	*p*-value
Psychiatric disorder	No (N=6)	14.768 [6.480-23.057]	0.008	1,36	0.929
	Yes (N=33)	16.524 [11.709-21.339]			
Major depressive disorder	No (N=23)	15.511 [9.416-21.606]	2.830	1,36	0.101
	Yes (N=16)	16.556 [10.914-22.197]			
Anxiety disorder	No (N=14)	15.553 [10.416-20.691]	0.311	1,36	0.581
	Yes (N=25)	17.114 [9.738-24.491]			
Psychotropic medication	No (N=6)	16.111 [5.561-26.660]	0.434	1,36	0.514
	Yes (N=36)	16.067 [11.570-20.564]			
Antidepressants	No (N=25)	13.047 [7.879-18.216]	0.090	1,36	0.766
	Yes (N=17)	20.424 [14.217-26.631]			
Anxiolytics	No (N=16)	12.289 [5.572-19.005]	0.464	1,36	0.500
	Yes (N=26)	18.193 [13.167-23.219]			
